# A living enzymatic adenosine switch couples metabolic rescue to IL-12-driven antitumor immunity

**DOI:** 10.21203/rs.3.rs-11368155/v1

**Published:** 2026-10-29

**Authors:** Stephanie Sendker, Shaobo Yang, Eden Bobilev, Alice Chen, Fuguo Liu, Anna Clara Bader, Xingyu Deng, Jenny Ho, Maily Nguyen, Michael J. James, Veronica W Hui, Kate Marinchev, Andreia Maia, Castilleja L Albert, Isabella Salinas, Ashley Hu, Alaa Kassim Ali, Jacqueline S. Garcia, Daniel DeAngelo, Mohammad Rashidian, Bartosz J. Grzywacz, Robert J. Soiffer, Jerome Ritz, Catherine J. Wu, John Koreth, Remy Dulery, Jose A. Cancelas, Roman Shapiro, Mounica Vallurupalli, Jiahe Li, Andrew A. Lane, Mubin Tarannum, Rizwan Romee

**Affiliations:** 1 Department of Medical Oncology, Dana-Farber Cancer Institute, Harvard Medical School, Boston, MA, USA; 2 Department of Pediatric Hematology and Oncology, West German Cancer Center, University Medicine Essen, Germany; 3 Parker Institute for Cancer Immunotherapy, San Francisco, CA, USA; 4 Faculty of Medicine, Julius-Maximillians University of Würzburg, Germany; 5 Department of Microbiology, Harvard Medical School, Boston, MA, USA; 6 Department of Pathology, Beth Israel Deaconess Medical Center, Boston, MA, USA; 7 Cancer Program, Broad Institute, Cambridge, MA, USA; 8 Department of Biomedical Engineering, University of Michigan, Ann Arbor, MI, USA

## Abstract

Cancer cells remodel their metabolic environment to disable infiltrating immune cells. Adenosine is a principal mediator of this immunosuppression, impairing the metabolism and effector functions of T and natural killer (NK) cells. Blocking adenosine receptors or production within a redundant signaling network leaves the metabolite itself intact and has shown limited clinical efficacy. Adenosine deaminase (ADA) instead converts adenosine into inosine, a metabolite that can fuel T cell metabolism. We engineered non-pathogenic, tumor-tropic *Escherichia coli* K-12 DH5α to surface-display ADA, confining this metabolic switch to the tumor, and co-displayed interleukin-12 (IL-12) to couple metabolic rescue to local immune stimulation. Bacteria-displayed ADA alone restored metabolic and proliferative fitness in adenosine-suppressed lymphocytes to baseline, whereas incorporation of IL-12 coupled this rescue to enhanced effector function. Across multiple preclinical models of hematologic and solid malignancies, the dual-function bacteria were well tolerated, enhanced tumor control, prolonged survival, and induced durable protective immunity against melanoma rechallenge. The bacteria were enriched at tumor sites, reactivated quiescent immune compartments in leukemia, enhanced CAR-NK cell tumor trafficking and activity in pancreatic cancer, and outperformed recombinant proteins in breast cancer. These findings establish a modular living immunotherapeutic strategy that couples a local extracellular metabolic switch with localized cytokine delivery.

## Introduction

Despite major advances in cancer immunotherapy, clinical success remains constrained by tumor-driven metabolic remodeling of the immune environment, fostering immunosuppression that undermines the persistence, metabolic fitness and antitumor function of immune-effector cells ^1–3^. Within this hostile environment, cellular stress, tissue damage and hypoxia drive extracellular purine catabolism and the accumulation of adenosine (ADO), a potent immunosuppressive metabolite that allows tumors to co-opt a physiological tissue-protective pathway to evade immune surveillance ^4–6^. Mechanistically, ADO conveys its immunosuppressive signaling through the G protein–coupled A2A and A2B receptors, impairing T- and NK-cell effector functions and promoting myeloid-mediated immunosuppression ^4,7,8^. Beyond canonical receptor-mediated suppression, ADO can directly constrain lymphocyte metabolism, further compromising immunecell fitness and antitumor function ^9^.

Efforts to counter adenosine-mediated immunosuppression have largely focused on limiting ADO production by inhibiting the ectoenzymes CD39 or CD73 or blocking downstream A2A-receptor signaling ^10–13^. However, these approaches target individual components within a redundant network rather than eliminating ADO itself and have so far failed to achieve broad success in early clinical trials ^14^. Harnessing the non-redundant enzymatic step of adenosine deaminase 1 (ADA1) to convert ADO into inosine (INO), offers a comprehensive strategy to simultaneously remove immunosuppressive ADO and generate INO, a metabolite with emerging immune-stimulatory properties. Under glucose restriction, this ADO-derived degradation product can fuel T cell metabolism as an alternative carbon source, promote antitumor T cell responses, and induce metabolic and epigenetic programs that have been shown to enhance CAR-T cell stemness and function ^15–17^. Clinically, the therapeutic use of ADA1 is established through pegylated ADA1 enzyme replacement, and gene-corrected hematopoietic stem cell transplantation enabling rapid metabolic detoxification in ADA1-deficient severe combined immunodeficiency (ADA-SCID), an inherited life-threatening disease characterized by profound T-, B-, and NK cell lymphopenia resulting from the accumulation of toxic purine metabolites ^18,19^.

Translating ADA as a metabolic strategy to cancer immunotherapy presents an opportunity to combine adenosine depletion with complementary immune stimulation. IL-12 provides such a complementary signal by potently activating T- and NK cell effector responses, but systemic administration of this cytokine is limited by a narrow therapeutic window and severe inflammatory toxicity ^20–22^. The central challenge is to develop a strategy to engineer metabolic rescue and complementary pleiotropic immune stimulation in the tumor microenvironment. Engineered non-pathogenic *Escherichia coli (E. coli)* offer such spatial control through their innate tumor tropism, ease of genetic programmability, accessibility and low cost ^23–28^.

We hypothesized that redirecting (rescue) ADO-driven metabolic pressure through tumor-localized ADA activity, while simultaneously providing IL-12-mediated immune stimulation, could rewire the tumor metabolic environment to restore immune-cell fitness and antitumor function. Here, we engineered non-pathogenic *E. coli* K-12 DH5α to surface-display catalytically active ADA1 (ADA from now onwards) with the potent immune-stimulating cytokine IL-12, generating a dual-function immune enhancing metabolic switch that reversed ADO-mediated metabolic and proliferative suppression and enhanced antitumor immunity across leukemia and solid-tumor models. We further benchmark bacteria displayed ADA–IL-12 against recombinant IL-12 and ADA proteins and combine the immunometabolic platform with mesothelin-targeted CAR-NK cell therapy, demonstrating that the living platform enhances the infiltration and efficacy of cellular therapy.

## Results

### *E. coli* surface-displaying ADA reverses adenosine-mediated metabolic suppression and restores immune-cell fitness

To overcome the adenosine metabolic checkpoint and locally convert adenosine (ADO) to inosine (INO), we engineered tumor-tropic, non-pathogenic *E. coli* to surface-display human ADA (hADA) or murine ADA (mADA) using the two N-terminal scaffolds YiaT181 and YiaT232 (Fig. 1a and Extended Data Fig. 1a). Flow cytometry confirmed surface expression of all four DYKDDDDK (FLAG)-tagged constructs (Fig. 1b, Extended Data Fig. 1b, Supplementary Fig. 1). Targeted liquid chromatography–mass spectrometry (LC–MS) of bacterial supernatants 1 and 3 hours after ADO supplementation showed progressive ADO depletion and conversion to INO, with mADA-displaying *E. coli* outperforming their hADA counterparts with both scaffolds (Fig. 1c and Extended Data Fig. 1c,d). YiaT181-mADA achieved the highest INO/ADO ratio, followed by YiaT232-mADA (Extended Data Fig. 1d). Enzyme kinetic analysis similarly demonstrated greater catalytic activity of murine than human ADA with both scaffold constructs with YiaT181-mADA showing the highest conversion rates (Extended Data Fig. 1e). YiaT181-mADA (E. ADA) was therefore selected for subsequent studies.

In primary human T cells and NK cells, ADO markedly impaired mitochondrial respiration (Fig. 1d,f). E. ADA reversed this suppression, restoring maximal oxygen consumption rate (OCR) and spare respiratory capacity relative to ADO and scaffold controls in T cells (Fig. 1d) and NK cells (Fig. 1f). Consistent with these results, OXPHOS gene set profiles were enriched in E. ADA-treated T and NK cells compared to the scaffold control (Fig. 1e,g). MitoTracker Green staining further indicated increased mitochondrial mass following exposure to E. ADA in ADO-stressed T cells (Fig. 1h) and NK cells (Fig. 1i), exceeding levels observed in unstressed controls and comparable to the cells supplemented with INO (Extended Data Fig. 2a and 2b, respectively). In a complementary manner, ADO slightly reduced the extracellular acidification rate (ECAR) which was restored by E. ADA in both T and NK cells, although the corresponding differences in glycolytic capacity did not reach statistical significance (Extended Data Fig. 2c,d). In addition, E. ADA further promoted lymphocyte proliferation and activation, increasing Ki-67 and the high-affinity IL-2 receptor alpha-chain CD25 in both T and NK cells beyond control levels (Fig. 1j,l). This phenotype was accompanied by increased expression of the activation-associated receptors CD69 and NKG2D (Extended Data Fig. 2e,f), and broader immunophenotypic profiling revealed concordant modulation of activation and immune-regulatory markers in both lineages (Supplementary Figs. 2a-c and 3a-c). Consistent with this restoration of lymphocyte fitness, E. ADA rescued ADO-impaired NK- and CAR-T cell cytotoxicity to levels observed in the absence of ADO (Extended Data Fig. 3a and b). Together, these findings show that surface-displayed E. ADA alleviates ADO-mediated suppression, restoring metabolic and proliferative fitness, activation-associated phenotypes, and cytotoxic function in T and NK cells.

To understand the potential mechanisms, we performed transcriptomic profiling, which corroborated the metabolic, proliferative and activation-associated phenotypes. In E. ADA-exposed T cells, oxidative phosphorylation and proliferation-associated Hallmark pathways, including MYC targets V1 and V2, ranked among the most positively enriched gene sets relative to scaffold controls (Fig. 1k). In NK cells, E2F targets, G2M checkpoint and MYC target V1 showed the strongest positive enrichment, followed by allograft rejection and oxidative phosphorylation (Fig. 1m). Conversely, hypoxia, glycolysis and TNF–NF-κB signaling were among the most strongly negatively enriched gene sets in E. ADA relative to E. scf-exposed T and NK cells (Supplementary Fig. 4a). This reciprocal transcriptional pattern was also evident at the individual gene level, with differentially expressed genes distinguishing the E. ADA- and E. scf-associated states in both T and NK cells (Supplementary Fig. 4b). Consistent with these results, comparison of cells exposed to ADO or its ADA-generated product INO showed preferential enrichment of metabolic and proliferative programs with INO, dominated by MYC targets and oxidative phosphorylation in T cells and E2F/G2M-associated programs in NK cells, whereas ADO favored TNF–NF-κB and other inflammatory or stress-associated programs (Extended Data Fig. 4). Similarly, relative to untreated cells, ADO exposure enriched hypoxia and TNF–NF-κB signaling, whereas untreated cells showed relative enrichment in metabolic and proliferative programs (Supplementary Fig. 5).

### Co-displaying IL-12 with ADA overcomes the cytotoxic ceiling of metabolic rescue alone

Although E. ADA robustly reversed ADO-mediated suppression, restoring metabolic fitness, proliferative capacity and tumor-cell-directed cytotoxicity to unstressed control levels, it did not augment cytotoxic activity beyond this baseline (Extended Data Fig. 3). To further potentiate effector function, we hypothesized that complementing ADA-mediated metabolic remodeling with IL-12-mediated immune stimulation would enhance the cytotoxic activity of the immune effector cells. We therefore engineered bacteria to co-display ADA and IL-12, generating a dual-function immunometabolic fusion construct (E. ADA–IL-12) (Fig. 2a). Based on the preceding scaffold screen (Extended Data Fig. 1), YiaT181-mADA was selected for dual-display engineering, with human or murine IL-12 incorporated in alternative fusion configurations (Fig. 2a). Flow cytometry confirmed surface expression of the FLAG-tagged fusion proteins (Fig. 2b) and incorporation of IL-12 maintained time-dependent ADO-to-INO conversion (Fig. 2c), with independent enzyme-kinetic analyses confirming preserved ADA catalytic activity and comparable reaction velocities across the dual constructs (Supplementary Table 3, Supplementary Fig. 6a). These constructs retained strong IL-12 activity even at low bacteria-to-HEK-Blue reporter-cell ratios (1:100, 1:10) and were comparable to that of recombinant IL-12 (50 ng/ml) and the corresponding IL-12 alone displaying constructs (Fig. 2d and Supplementary Fig. 6b-d). Positioning IL-12 downstream of ADA yielded modestly greater ADO-to-INO conversion and IL-12 activity than the reciprocal configuration (Fig. 2c,d and Supplementary Fig. 6c,d). The YiaT181-displayed mADA–IL-12 configuration was therefore selected for all the subsequent experiments.

We next tested effector function *in vitro*. IL-12-displaying *E. coli* (E. IL-12) enhanced NK cell cytotoxicity against leukemic NOMO-1and pancreatic AsPC1 cells, as well as CD70 CAR-T cell-mediated cytotoxicity against OCI-AML3 cells (Fig. 2e, f and Extended Data Fig. 5a,b). Although E. IL-12 significantly increased tumor cell lysis relative to control conditions by NK- and CAR-T cells, this enhancement remained constrained under ADO stress (Supplementary Fig. 7a–c). However, *E. coli* co-displaying ADA and IL-12 (E. ADA–IL-12) overcame this constraint, significantly enhancing cytotoxicity of NK and CAR-T cells against hematologic and solid-tumor targets (Fig. 2e–g, Supplementary Fig. 8, Extended Data Fig. 5a,b). Under ADO-mediated suppression, E. ADA–IL-12 further increased target cell lysis beyond the DMSO control levels (Fig.2f and Extended Data Fig. 5a,b), accompanied by increased TNF, IFN-γ and CD107a expression, indicating enhanced effector cytokine production and degranulation (Fig. 2g and Extended Data Fig. 5a,b). Consistent with this enhanced effector cell function, immunophenotyping revealed a more activation-associated phenotype in T and NK cells after E. ADA–IL-12 stimulation (Supplementary Fig. 9). In T cells, E. ADA–IL-12 modestly increased CD25, CD69 and NKG2D relative to E. IL-12 or E. ADA (Supplementary Fig. 9a); Ki-67 was largely unchanged, whereas the immunoregulatory markers TIM-3 and PD-1 were modestly elevated relative to E. ADA but remained similar to E. IL-12. By contrast, CTLA-4 was reduced relative to E. IL-12 under ADO stress (Supplementary Fig. 9b). Similarly, in NK cells, E. ADA–IL-12 increased the expression of activation receptors NKG2D and NKp30, whereas CD25 and CD69 remained comparably high across E. ADA–IL-12, E. IL-12 and E. ADA conditions under ADO stress (Supplementary Fig. 9c). As in T cells, Ki-67 was comparable between the bacterial treatment groups, but higher with E. ADA–IL-12 than with recombinant IL-12. Immunoregulatory markers were largely unchanged across these conditions, except for modestly reduced KIR expression relative to the scaffold control (Supplementary Fig. 9d). Together, these findings demonstrate that IL-12 complements ADA-mediated metabolic and proliferative restoration with increased cytotoxic effector activity and a more activated phenotype, extending the functional cytotoxicity of NK- and CAR-T cell responses across hematologic and solid-tumor targets.

To assess the therapeutic activity of E. ADA–IL-12 *in vivo*, we first used a syngeneic B16-F10 melanoma model (Fig. 2h). Intratumoral treatment with both E. ADA and E. ADA–IL-12 significantly reduced tumor growth compared with the scaffold and PBS controls, with E. ADA–IL-12 providing the strongest tumor control and survival benefit, with four out of six mice surviving beyond 60 days after tumor implantation (Fig. 2i,j). To determine whether this response was durable, all long-term survivors were rechallenged with B16-F10 cells without additional treatment (Fig. 2k). While tumors rapidly progressed in treatment naïve mice, all previously treated survivors remained protected following rechallenge (Fig. 2l,m), consistent with the induction of durable antitumor immunity following E. ADA–IL-12 treatment.

To define the transcriptional programs underlying these effects, we profiled T and NK cells exposed to E. ADA–IL-12 and relevant controls. E. scf control exposure remodeled the purine–adenosine axis, characterized by reduced *ADA, PNP* and *DPP4* and increased *ADORA2B* expression in T and NK cells, with corresponding broader changes in adenosine-related surface markers (Extended Data Fig. 6a,b,e,f). E. scf exposure also induced strong enrichment of the Hallmark Hypoxia gene set in both cell types, which was reversed in E. ADA–IL-12 relative to E. scf (Extended Data Fig. 6c,d). In T cells, E. ADA–IL-12 enriched IFN-γ and IFN-α-response and allograft-rejection signatures relative to E. scf controls, alongside MYC-, E2F- and G2M-associated programs (Fig. 3a,b). Pathway-annotated gene expression further resolved this coordinated immune-effector and proliferative response: In T cells, E. ADA–IL-12 increased *HLA-DRA* and *CCL5* within the IFN-γ-response program, *CD3D, CD3E, CD247* and *HLA-DRA* within the allograft-rejection program, and MYC-target genes including *SRSF3, SRSF2, TRA2B* and *SRSF7* (Fig. 3c). Among the most strongly upregulated genes in *E.* ADA–IL-12-exposed T cells were the chemokine receptors *CCR5* and *CXCR6*, consistent with acquisition of an activated T cell state with enhanced migratory and tissue-trafficking potential (Fig. 3d). Conversely, E. scf-treated cells preferentially expressed inflammatory and stress-associated transcriptional programs, including TNF–NF-κB-, p53- and hypoxia-associated signatures, with *CXCL2* and *ATF3* among the prominent scaffold-enriched genes and additional increases in *MT1X* and *CLIC4* (Fig. 3c,d). Together, these data show that E. ADA–IL-12 induced a coordinated IFN-associated immune-effector state with allograft-rejection and chemokine-receptor remodeling features while preserving the ADA-associated MYC-, E2F- and G2M proliferative transcriptional programs. This contrasts with the predominantly stress-inflammatory gene sets in E. scf exposed T cells (Supplementary Fig. 10a).

A related but more prominently proliferative transcriptional response was observed in NK cells, with E. ADA–IL-12 enriching E2F, G2M and MYC alongside oxidative phosphorylation hallmark gene sets, while allograft-rejection and IFN-γ signatures indicated concurrent immune-effector activation compared with E. scf controls (Fig. 3e). Enrichment profiles confirmed representative E2F, allograft-rejection and IFN-γ responses (Fig. 3f). At the individual gene level, E. ADA–IL-12-treated NK cells showed broad induction of cell-cycle (*RPS6, TFRC*), mitotic (*TUBB, TUBA1B*) and metabolic pathway-associated (*EEF1A1, EEF2*) genes together with an immune-response component (*IL2RB, CD2),* whereas E. scf-treated cells displayed a more stress-associated (*HSPA8, HSP90AA1*) and inflammatory gene pattern (*JUN, FOS*) (Fig. 3g), consistent with preferential enrichment of hypoxia, UPR and TNF–NF-κB signatures in E. scf treated NK cells (Supplementary Fig. 10a). Differential gene expression further supported the dominant proliferative phenotype, with increased expression of DNA-replication and mitotic regulators including *MCM5, KNL1, DTL, MYBL2, KIFC1 and SPC25* (Fig. 3h). These proliferative and metabolic programs recapitulated the transcriptional phenotype induced by E. ADA alone (Fig. 1), whereas the accompanying allograft-rejection and IFN response pattern revealed an additional immune-effector component of the dual construct. Additional direct comparison of the more subtle transcriptional differences between E. ADA–IL-12 and E. ADA exposed NK and T cells identified additional cytokine- and activation-associated pathways in T and NK cells following IL-12 incorporation (Supplementary Fig. 10b). Together, these data show that E. ADA–IL-12 couples ADA-associated metabolic and proliferative response with a prominent immune-effector program, providing a transcriptional basis for enhanced IFN-γ/TNF production and cytotoxicity observed with the dual construct.

### E. ADA–IL-12 remodels the immune environment and induces potent antileukemic responses in adenosine-rich AML

To extend the dual metabolic–cytokine ADA–IL-12 strategy to a systemic hematologic malignancy, we first evaluated the clinical relevance of the adenosine axis in AML. Patients with AML had higher bone marrow ADO levels at initial diagnosis (n=10) than at remission (n=4), with a modest increase also observed in peripheral blood (Fig.4a and Extended Data Fig. 7a,b). Moreover, ADO levels positively correlated with leukemic blast burden in both bone marrow (Fig. 4b) and peripheral blood (Extended Data Fig. 7c). These findings establish elevated adenosine as a feature of active AML and provide a rationale for us to locally target this axis with ADA–IL-12 in a systemic disease setting.

Given this clinical relevance of the adenosine metabolic axis in AML, we systematically evaluated E. ADA–IL-12 in a syngeneic WEHI-3B AML model. Mice received two intravenous (tail vein) injections of bacteria on days 5 and 10 after leukemia inoculation (Fig. 4c). The adenosine concentrations in E. ADA and E. ADA–IL-12-treated animals were significantly lower compared to the E. IL-12 and E. scf controls (Extended Data Fig. 7d,e), confirming functional ADA activity *in vivo*. Notably, GFP^+^ WEHI-3B leukemia cells preferentially accumulated in the liver and spleen across all treatment groups. The leukemia burden (assessed by frequency of GFP^+^ cells) was markedly decreased following E. ADA–IL-12, E. ADA and E. IL-12 treatment relative to PBS and E. scf controls (Fig. 4d, Supplementary Fig. 11a and 12). Importantly, PBS- and E. scf-treated mice rapidly succumbed to disease, with a median survival of 13 days, whereas E. ADA–IL-12 extended median survival to 38 days, outperforming E. ADA or E. IL-12 alone (Fig. 4e and Supplementary Fig. 11b).

In parallel, bacterial biodistribution revealed highest bacterial loads in the liver at 2 hours and on day 4 after the last (day 10) intravenous bacterial injection (Fig. 4f). At 2 hours, bacteria were detectable in BM, peripheral blood, spleen, and liver, with hepatic recovery being highest in E. ADA–IL-12 (E. ADA–IL-12, 1.9×10^9^ CFU g^−1^ versus E. scf, 1.6×10^7^ CFU g^−1^). Bacterial burden rapidly declined across tissues in all treatment conditions by day 4 with residual bacteria remaining detectable only in the spleen and liver. Complementary immunohistochemical analysis of bone-marrow sections 2 hours after the second day 10 bacterial injection revealed intracellular bacterial localization within neutrophilic granulocytes, consistent with phagocytic uptake and possible lysosomal compartmentalization (Extended Data Fig. 7f). Notably, IHC staining for the FLAG tag showed fewer bacteria than staining against native *E. coli*, potentially reflecting reduced expression or degradation of the engineered protein following phagocytic uptake.

UMAP analysis of immune-phenotyped liver samples resolved a prominent granulocyte-enriched population separated from a myeloid enriched cluster with distinct DC-like, monocyte- and macrophage-enriched populations (Fig. 4g). Consistent with preferential hepatic bacterial accumulation, E. ADA–IL-12-treated mice showed significantly increased hepatic CD45^+^ leukocytes (Fig. 4h), together with pronounced myeloid remodeling featured by increased DC-like, XCR1^−^ cDC2-like populations and enriched myeloid cells in E. ADA- and E. ADA–IL-12-treated mice, with most prominent increase in macrophage-enriched cells (Fig. 4h and Supplementary Fig. 13a). Peripheral-blood profiling revealed similarly selective remodeling of the myeloid compartment. UMAP analysis resolved a distinct myeloid compartment comprising DC-like, monocyte- and macrophage-enriched populations, separated from the major lymphoid and granulocytic populations (Supplementary Fig. 13 b,c). Although granulocytes constituted a major fraction of circulating leukocytes and remained largely unchanged across treatment groups, E. ADA–IL-12 increased DC-like, monocyte- and macrophage-enriched populations without substantially altering overall CD45^+^ leukocyte abundance (Supplementary Fig. 13c). Bone-marrow UMAP delineated a predominant, separated granulocyte population, a moderately represented lymphoid compartment with closely associated T- and B-cell populations, and a comparatively smaller myeloid compartment than in other tissues (Supplementary Fig. 13d). Bacteria-treated bone marrow samples showed significantly increased granulocyte and myeloid cell populations, whereas E. ADA–IL-12 selectively increased DC-like cells including cDC1- and cDC2-subpopulations, a pattern that partly mirrors the myeloid remodeling observed in the liver (Supplementary Fig. 13e). Splenic UMAP showed prominent T- and B-cell populations, alongside a smaller, phenotypically interconnected myeloid compartment and a separate granulocyte population (Supplementary Fig. 13f). Quantitative analysis of spleen samples identified a predominantly E. scf-driven increase in granulocytes, which was attenuated in ADA-exposed conditions. DC-like cells were enriched in ADA-exposed samples, while myeloid cell populations increased particularly following treatment with bacterial scaffold, E. IL-12 and E. ADA (Supplementary Fig. 13g). Overall, bacterial exposure induced tissue-dependent granulocytic and myeloid remodeling, whereas ADA-containing treatments more selectively enriched DC-like populations, with limited changes in major lymphocyte populations.

Despite this treatment-associated immune remodeling across tissues, key cytokine-release syndrome (CRS)-associated cytokines, including IL-6, IL-1β, TNF and GM-CSF remained low in blood and bone marrow across all treatment groups, without reaching concentrations typically associated with clinically apparent systemic inflammation (Fig. 4i and Supplementary Fig. 14). Within this overall low cytokine profile, E. ADA–IL-12 treatment was associated with increased IFN-γ and IL-2 levels compared with those in E. scf and PBS-treated control mice (Fig. 4i). Broader profiling of inflammatory-linked cytokines and chemokines showed no indication of systemic inflammatory responses (Supplementary Fig. 14). Together with stable body weight (Supplementary Fig. 15) and the absence of overt treatment-related toxicity in this immunocompetent model, these findings indicate that repeated systemic E. ADA–IL-12 administration was well tolerated, coupling selective immune activation with limited systemic inflammation.

Collectively, these data indicate that systemic E. ADA–IL-12 drives tissue-selective immune remodeling and antileukemic activity with limited systemic inflammation and good tolerability under the tested treatment schedule, resulting in reduced leukemic burden and prolonged survival in WEHI-3B AML bearing mice.

### E. ADA–IL-12 enhances antitumor efficacy across solid tumors, potentiates CAR-NK cell therapy and outperforms recombinant protein therapy

Building on the efficacy and tolerability of E. ADA–IL-12 in the WEHI-3B AML model, we extended this strategy to solid tumors, to evaluate its potential to overcome the highly immunosuppressive tumor environment and limited immune-cell trafficking, two key barriers to effective cellular therapy.

Peripheral blood-derived NK cells were transduced with a second-generation 4-1BB/CD3ζ-based mesothelin (MSLN) CAR using an optimized baboon-envelope lentiviral protocol (Supplementary Fig. 16) ^29^. Treatment with E. ADA–IL-12 significantly increased MSLN CAR-NK cell cytotoxicity against AsPC1 PDAC cells *in vitro*, extending the enhanced cytotoxic activity observed with untransduced NK cells (Extended Data Fig. 8a,b). We next evaluated this combination in NSG mice bearing subcutaneous AsPC1 xenografts. Mice received two intravenous infusions of MSLN CAR-NK cells (5×10^6^) on days 18 and 32 with intravenous PBS, E. scf or E. ADA–IL-12 administration on days 18, 25, 32 and 39 after tumor implantation (Fig. 5a). E. ADA–IL-12 markedly enhanced CAR-NK-mediated tumor control relative to PBS and scaffold controls (Fig. 5b) and significantly extended median survival from 51 days (MSLN CAR-NK plus PBS or E. scf) to 77 days (Fig. 5c). Biodistribution analysis demonstrated preferential enrichment of intravenously administered bacteria within AsPC1 tumors relative to non-tumor organs as assessed on day 36 (Fig. 5d). Intratumoral adenosine concentrations were reduced following E. ADA–IL-12 treatment relative to scaffold controls, consistent with tumor infiltration and functional ADA activity of the injected bacteria *in vivo* (Extended Data Fig. 8c). This metabolic remodeling was accompanied by increased intratumoral human CD45^+^ cell abundance (Extended Data Fig. 8d, Supplementary Fig. 17) and increased accumulation of GFP^+^ MSLN CAR-NK cells in E. ADA– IL-12 treated mice (Fig. 5e and Extended Data Fig. 8e). The increased abundance of human CD45^+^GFP^+^ cells in tumors from E. ADA–IL-12-treated animals coincided with higher intratumoral bacterial CFU compared with scaffold-treated controls. Fluorescence microscopy on day 28 further confirmed bacterial localization within tumors, with E. ADA–IL-12 preferentially detected in regions of reduced nuclear staining, consistent with accumulation in low-cellularity or necrotic tumor areas (Fig. 5f). Notably, the body weight remained stable across treatment groups and throughout the treatment period, supporting tolerability of repeated systemic MSLN CAR-NK and bacteria co-administration (Extended Data Fig. 8f). Together, these findings demonstrate that E. ADA–IL-12 preferentially localizes to PDAC tumors, where it depletes intratumoral adenosine and enhances tumor infiltration of and tumor control by MSLN CAR-NK cells, supporting tumor-localized metabolic and immune modulation as a strategy to overcome key barriers to cellular therapy in solid tumors.

Lastly, we asked if the living bacterial platform would provide a therapeutic advantage beyond the delivery of its corresponding soluble components and hence compared E. ADA–IL-12 with soluble ADA and recombinant IL-12 in the immunocompetent orthotopic 4T1 mammary carcinoma model (Fig. 6a). E. ADA–IL-12 treatment resulted in sustained tumor control with significantly reduced tumor growth compared with PBS and scaffold control animals (Fig. 6b). Similarly, the protein combination of soluble ADA and IL-12 reduced tumor growth, although to a lesser extent than E. ADA–IL-12 (Fig. 6b, Supplementary Fig. 18). Both protein ADA plus IL-12 and bacteria-displayed ADA–IL-12 improved survival compared with PBS and scaffold controls (Fig. 6c). However, bacteria-displayed ADA–IL-12 conferred significantly longer survival than the recombinant protein combination (Fig. 6c). Consistent with these differences in tumor growth and survival, endpoint quantification of mCherry^+^ cells in isolated tumor tissues showed a significantly lower tumor burden following E. ADA–IL-12 treatment compared with PBS and scaffold controls. Importantly, mCherry^+^ tumor-cell burden was also significantly lower with bacteria-displayed ADA–IL-12 than with the soluble protein ADA plus IL-12 combination (Fig. 6d). The superior therapeutic activity of bacteria-displayed ADA–IL-12 in this intratumorally treated orthotopic model indicates a benefit of bacterial delivery beyond its tumor-tropism alone. The enhanced antitumor activity was accompanied by remodeling of the intratumoral immune compartment, with increased CD3^+^ T cells in both recombinant protein- and bacteria-displayed ADA–IL-12-treated tumors and enrichment of CD11b^+^ myeloid and DC-like populations, recapitulating aspects of the myeloid remodeling pattern observed across tissues in the WEHI-3B AML model (Fig. 6e). Broader immunophenotyping further identified bacteria-associated increase of granulocyte and CD8^+^ T cell populations within treatment-associated immunophenotypic changes (Supplementary Fig. 19). Together, these findings demonstrate greater therapeutic activity of living bacterial ADA–IL-12 display compared with administration of the corresponding recombinant proteins in an orthotopic treatment setting, indicating therapeutic benefit of bacterial delivery beyond tumor tropism and coupling improved tumor control and survival with remodeling of the endogenous antitumor immune compartment in an immunocompetent solid-tumor model.

## Discussion

We establish a living therapeutic strategy that couples enzymatic remodeling of the tumor metabolome to localized immune stimulation. Rather than blocking components of a redundant ADO pathway leaving the suppressive metabolite intact, ADA surface displayed by tumor homing nonpathogenic *E. coli* locally remodels the extracellular tumor metabolome, replacing immunosuppressive ADO with INO, thus enhancing metabolic and proliferative fitness of suppressed T and NK cells and turning a metabolic checkpoint into a source of metabolic support. Metabolic rescue alone, however, restored cytotoxicity only to baseline. Co-displaying IL-12 on the same bacterium broke that ceiling, and the relationship proved reciprocal; IL-12 activity was itself constrained under adenosine-rich conditions, and ADA relieved that constraint. Coupling the two functions on a single tumor-homing chassis therefore produced an effect that neither payload achieved alone.

Surface-displayed ADA efficiently depleted ADO, which is a potent immunosuppressive metabolite that constrains immune-cell function ^5,30–32^. Consistent with previous studies, our data substantiate the inhibitory effects of ADO on T and NK cell immunity with E. ADA-mediated restoration of proliferation, activation and metabolic capacity. At the transcriptional level, these E. ADA-mediated pro-metabolic and proliferative phenotypes were mirrored by enrichment of OXPHOS and MYC programs in T and NK cells, with additional E2F and G2M cell-cycle enrichments in NK cells, suggesting particularly pronounced cell-cycle engagement in NK cells. Consistent with the restored immune-activation phenotype seen in E. ADA-treated T and NK cells, associated gene sets such as allograft-rejection were enriched in E. ADA relative to scaffold, further reflecting this phenotype at the transcriptional level. While INO exposure alone restored oxidative capacity and partially recapitulated ADA-associated immunophenotypic changes, it did not reproduce the full E. ADA phenotype. Transcriptionally, E. ADA most closely reflected the ADO-to-INO transition across T and NK cells, whereas neither INO nor reciprocal ADO effects alone fully recapitulated its transcriptional state. Thus, ADA-mediated recovery appears to integrate ADO depletion with generation of the enzymatic product INO. This finding builds on previous studies establishing INO as an active purine metabolite that supports T cell central carbon metabolism under nutrient-restricted conditions, enhances antitumor T cell responses and promotes CAR-T cell metabolic fitness and potency ^15–17^. Our findings extend this biology to NK cells and identify ADA-mediated ADO-to-INO conversion as an efficient metabolic switch strategy to combine enzymatic conversion of an immunosuppressive metabolite with the generation of a metabolically supportive product.

Although E. ADA induced a metabolic ADO-to-INO switch, cytotoxic activity was restored only to baseline levels. Adding IL-12 overcame this ceiling, while IL-12 activity itself was constrained under ADO-rich conditions, and ADA relieved this constraint, creating a mutually reinforcing interaction in which metabolic rescue unleashes IL-12’s effector potential. E. ADA–IL-12 largely preserved the ADA-associated transcriptional pattern, with metabolic and proliferative programs remaining among the dominant positively enriched gene sets relative to scaffold-exposed cells, highlighting restored immune-cell fitness as a major component of the response to both ADA-containing constructs. Notably, direct comparison with E. ADA uncovered additional TNF–NF-κB and IL-2–STAT5 signaling, consistent with previous studies linking IL-12-containing cytokine stimulation to enhanced cytokine responsiveness and cytotoxic function ^33,34^. The greater cytotoxic activity of E. ADA–IL-12 therefore coincided with a comparatively focused inflammatory and cytokine-responsive program layered onto the dominant ADA-mediated metabolic response. Notably, this remodeling also corrected an unexpected, hypoxia-associated ADO-favoring shift in the purine-adenosine axis (reduced ADA/PNP/DPP4, increased ADORA2B) ^35,36^ induced by scaffold bacteria themselves. This parallel regulation of hypoxia and ADO-purinergic programs implicates a previously unrecognized hypoxia-driven link between bacterial scaffold exposure and purine–adenosine remodeling as additional source of environmental ADO-pressure that E. ADA–IL-12 fully reversed. These mechanisms translated into potent antitumor activity across diverse settings. In syngeneic B16-F10 melanoma, E. ADA alone prolonged survival, showing that this metabolic switch alone can already be effective, while E. ADA–IL-12 conferred greater benefit, with long-term survivors rejecting tumor rechallenge beyond 60 days, consistent with protective immunological memory and underscoring additional advantage of coupling metabolic recovery with complementary effector stimulation. Extending this platform to hematologic malignancy we observed elevated ADO in patients with AML at initial diagnosis, and these elevated ADO levels correlated with leukemia blast burden in blood and bone marrow making a case for evaluating our metabolic–cytokine approach in acute leukemia. In an aggressive syngeneic WEHI-3B AML model, systemic administration of E. ADA–IL-12 depleted extracellular ADO, reduced tumor burden across blood, bone marrow, spleen and liver, and prolonged survival beyond either E. ADA or E. IL-12 alone. Bacterial localization matched the leukemic distribution (highest in liver and spleen) ^37^, with ADA-expressing bacteria recovered at higher levels than scaffold, suggesting enhanced tropism towards leukemia-enriched tissues. Notably, a shift of the tolerogenic liver toward an immune-active state including myeloid remodeling of monocyte/macrophage, and DC-like populations persisted across compartments alongside a broader bacteria-associated increase in granulocytes in liver, spleen and bone marrow even after the bacteria became undetectable. In the absence of systemic toxicity or weight loss, these findings suggest that transient exposure may suffice to initiate a sustained, well-tolerated immune activating response. The platform further potentiated adoptive cell therapy, particularly CAR-NK cell-based approaches, whose efficacy in solid tumors is often constrained by limited tumor infiltration and an immunosuppressive environment. In the AsPC1 PDAC xenograft model, E. ADA–IL-12 improved MSLN CAR-NK cell mediated tumor control and survival while also increasing intratumoral CAR-NK cell trafficking, a central barrier to adoptive cell therapy in solid tumors. Consistent with previous CD73–ADO axis targeting approaches that enhance NK cell infiltration and activity ^38,39^, this benefit coincided with reduced ADO and preferential bacterial accumulation, suggesting that tumor-localized E. ADA–IL-12 activity created a microenvironment more permissive to CAR-NK cell accumulation and function. Our findings extend this principle by using a tumor-localizing bacterial platform to combine enzymatic remodeling of extracellular ADO with cytokine-mediated immune stimulation, thereby enhancing the intratumoral abundance and therapeutic efficacy of antigen-specific CAR-NK cells.

Finally, in the immunocompetent orthotopic 4T1 breast cancer model, E. ADA–IL-12 achieved greater tumor control and survival than the equivalent protein ADA plus IL-12. This direct comparison demonstrates that therapeutic efficacy is not determined by payload identity alone and highlights bacterial surface display and delivery as an integral component of the therapeutic response. Our data indicate that this advantage over soluble proteins extends beyond the tumor-tropic properties of bacterial delivery alone and further suggests an additional contribution from bacteria-mediated immune activation and tumor-immune modulation. Prominent remodeling of DC-like populations following both bacterial and protein ADA and IL-12 treatment partially recapitulates the myeloid immune signature observed across systemic leukemia compartments suggesting a relevant contribution of dendritic-cell-mediated immune remodeling to the observed therapeutic benefit. The superior activity of bacteria-displayed ADA–IL-12 may therefore reflect additional advantages conferred by bacterial delivery beyond tumor localization, together with sustained extracellular enzymatic activity and spatial coordination of ADA and IL-12, although the relative contribution of these mechanisms remains to be determined. Together, these findings support the efficacy and tolerability of the bacterial approach in an immunocompetent orthotopic solid-tumor setting.

Despite these promising *in vitro* and *in vivo* results, the relative contributions of tumor localization, sustained extracellular enzymatic activity, spatial coordination of ADA and IL-12, and Toll-like receptor (TLR) agonism by the bacterial outer membrane remain to be defined. Surface display may further contribute to sustained ADA activity by stabilizing the enzyme at the bacterial envelope and limiting its susceptibility to proteolytic degradation, as described for bacterial surface-displayed enzymes ^40,41^. Whether YiaT181-mediated surface display similarly stabilizes ADA in vivo, and thereby contributes to sustained adenosine depletion, remains to be determined. The preclinical models used here also have limitations, particularly the xenograft model used for the CAR-NK cell experiments, warranting further validation in complementary models, including patient-derived xenografts. Finally, clinical translation of a live bacterial therapeutic will require evaluation of biological containment, dose and schedule optimization, and host immunogenicity, particularly antibacterial immunity that may limit repeated dosing.

Together, our results establish a modular living therapeutic strategy that spatially couples extracellular metabolic remodeling with cytokine-mediated immune stimulation at tumor sites, enabling durable activity across hematologic and solid tumors with favorable tolerability. Beyond ADA–IL-12, the ability to display functional proteins on the bacterial surface enables metabolic enzymes and immune effectors to be rationally combined according to the distinct metabolic and immune barriers of a given disease. Thus, this platform provides a versatile framework for localized remodeling of the tumor microenvironment and simultaneous targeting of the complementary mechanisms of tumor immune escape.

## Methods

### Ethics

All studies complied with relevant ethical regulations. Animal studies were performed in accordance with federal, state and institutional guidelines and were approved by the Institutional Animal Care and Use Committee of Dana-Farber Cancer Institute (DFCI; protocol 24-009). De-identified leukapheresis collars from anonymous healthy donors were obtained from the Crimson Core at Brigham and Women’s Hospital (resource identifier T0197). Blood and bone marrow biospecimens were collected from patients with AML who were consented to DFCI protocol #01-206 in accordance with the Declaration of Helsinki.

### Cell culture

Cell lines, sources and culture conditions are listed in Supplementary Table 1. Mammalian cells were maintained at 37 °C with 5% CO_2_. Unless otherwise indicated, media were supplemented with 1% penicillin–streptomycin. Cell lines were passaged at approximately 80% confluence. Cultures were routinely tested and confirmed negative for mycoplasma contamination.

### Generation of fluorescently labelled cell lines

The NanoLuc–GFP and firefly luciferase-mCherry lentiviral plasmids were generated using NEBuilder HiFi DNA Assembly (New England Biolabs). WEHI-3B cells were transduced with NanoLuc–GFP, and OCI-AML3, AsPC1, 4T1 and NOMO-1 cells with firefly luciferase–mCherry. Cells were transduced in the presence of polybrene (Sigma-Aldrich), and medium was replaced 24 h later. Transduced cells were enriched by fluorescence-activated cell sorting (FACS), gating on viable GFP^+^ or mCherry^+^ expression.

### Primary human T- and NK cell isolation and culture

Peripheral blood mononuclear cells (PBMCs) were isolated from healthy-donor leukapheresis collars by Ficoll-Paque density-gradient centrifugation. T cells were enriched using the EasySep Human CD3 Positive Selection Kit II (STEMCELL Technologies) and activated in TexMACS medium containing 50 U ml−^1^ recombinant human IL-2 and TransAct reagent (1:100, Miltenyi Biotec, 130-111-160). After 3 days, cells were washed and maintained in TexMACS medium containing 50 U ml^−1^ IL-2. NK cells were enriched using the RosetteSep Human NK cell Enrichment Kit (STEMCELL Technologies) and maintained in NK MACS medium containing 5% human serum and 500 U ml^−1^ recombinant human IL-2. Experimental NK- and T-cell cocultures lasting <48 h were performed in RPMI medium supplemented with 5% human serum and 5 ng ml^−1^ recombinant human IL-15. For cocultures exceeding 48 h (those performed prior to RNA sequencing) cells were maintained in NK MACS medium or TexMACS medium, as appropriate for NK and T cells, respectively, supplemented with 5% human serum and 5 ng ml^−1^ recombinant human IL-15.

### Generation of CAR-T and CAR-NK cells

CD70 CAR-T cells were generated from primary human T cells. T cells were transduced 2–3 days after activation with a lentiviral vector encoding a 2^nd^ generation CD70-specific CAR comprising the ectodomain of CD27 as the binding domain, 4-1BB/CD3ζ signaling domains, and GFP reporter. Following transduction, cells were expanded in TexMACS medium supplemented with 50 U ml^−1^ IL-2. Transduction efficiency was determined by GFP expression using flow cytometry. CAR-T cells were used for functional experiments 7–14 d after transduction. Mesothelin (MSLN) CAR NK cells were generated as previously described ^29^. For CAR-NK generation, NK cells were preactivated with IL-12 (10 ng ml^−1^), IL-18 (50 ng ml^−1^) and IL-15 (50 ng ml^−1^i) for 12–18 hours, washed and rested for 24 h before transduction using baboon envelope pseudotyped lentivirus (BaLV). The MSLN-CAR encoded a YP218-derived scFv, CD8 hinge and transmembrane domains, 4-1BB and CD3ζ signaling domains, and an eGFP reporter separated by P2A. For transduction, RetroNectin-coated plates (20 μg ml^−1^) and Vectofusin-1 (10 μg ml^−1^) were used, followed by spinoculation at 4,000 r.p.m. for 90 min. Cells were subsequently cultured in NK MACS medium supplemented with 500 U ml^−1^ IL-2. Transduction efficiency was quantified by eGFP expression.

### Bacterial culture and surface-display constructs

Non-pathogenic *E. coli* K-12 DH5α carrying the respective surface-display plasmids were maintained in lysogeny broth (LB) containing 50 μg ml^−1^ kanamycin. Overnight cultures were grown at 37 °C with shaking at 250 r.p.m. and diluted tenfold into fresh selective LB before induction. Bacterial concentrations were estimated by optical density at 600 nm (OD_600_), using OD_600_=8×10^8^ colony-forming units (CFU)ml^−1^.

### Plasmid construction and bacterial surface display

Surface-display constructs were generated using the rhamnose-inducible pDS861-derived plasmid backbone (Quintara Biosciences) incorporating either the YiaT181 or YiaT232 surface-display scaffold^27^. Human or murine adenosine deaminase (hADA or mADA) and IL-12 were fused to the C’ terminus of the respective YiaT display scaffolds through GGGGS linkers (3×GGGGS). DNA fragments were inserted C-terminally between the NotI and BamHI sites of pDS861-derived constructs using NEBuilder HiFi DNA Assembly Master Mix (New England Biolabs). Constructs incorporated DYKDDDDK (FLAG) and Myc epitope tags for detection of surface-displayed proteins. Final plasmids were validated by Sanger sequencing or nanopore-based whole-plasmid sequencing.

YiaT181-hADA, YiaT181-mADA, YiaT232-hADA and YiaT232-mADA were evaluated during initial surface-display screening, with YiaT181-mADA selected for subsequent *E.* ADA experiments. Bifunctional ADA–IL-12 constructs comprising mADA fused to human or murine IL-12 in both fusion orientations were generated analogously. YiaT181-displayed mADA–IL-12 was selected for subsequent *E.* ADA–IL-12 experiments. For induction, overnight bacterial cultures were diluted tenfold into fresh selective LB. pLyGo- and pDS861-derived constructs were induced with 10 mM L-rhamnose, whereas pDSG323-derived constructs were induced with 100 ng ml^−1^ anhydrotetracycline. Cultures were induced at 25 °C with shaking at 250 r.p.m. for 24 to 36 hours before collection.

### Bacterial surface-display analysis

Surface expression was quantified by flow cytometry using the incorporated epitope tag. Bacteria were washed with PBS, stained with anti-DYKDDDDK antibody (1:500 dilution, BioLegend) for 15 minutes at room temperature, washed and analyzed by flow cytometry. Expression was quantified relative to scaffold-only bacteria.

### Protein-structure prediction

Structures of the indicated ADA and ADA–IL-12 fusion proteins were predicted using AlphaFold version 2 ^42^ and visualized using open-source PyMOL v.3.1 (Schrödinger, LLC) installed through the Anaconda distribution.

### Adenosine and inosine quantification by LC–QTOF-MS

Adenosine (ADO) and inosine (INO) were quantified by high-resolution UHPLC–QTOF-MS at the Harvard Medical School Analytical Chemistry Core. Data were acquired and analyzed using Agilent MassHunter software, and metabolite concentrations were determined from calibration curves. For bacterial ADA activity assays, equivalent amounts of the indicated *E. coli* constructs were incubated with 1 mM ADO at 37 °C. Cell-free supernatants were collected at 0, 1 and 3 hours, precipitated with ice-cold acetonitrile-methanol and centrifuged at 12,000–16,000 × g for 15 minutes. Clarified extracts were diluted 1:500 in methanol and analyzed for ADO and INO. ADA-mediated conversion was expressed as the INO/ADO ratio. Mammalian culture supernatants and plasma samples were precipitated with ice-cold acetonitrile–methanol, centrifuged at 12,000–16,000 × g for 15 minutes and analyzed neat or after dilution in methanol.

### Colorimetric ADA activity assay

ADA activity was quantified using the Adenosine Deaminase/ADA Activity Assay Kit (Novus Biologicals, NBP3-24456) according to the manufacturer’s instructions. Soluble calf intestinal ADA (Roche, 10102105001; 0.5 U per reaction) was included as a positive reference control. Absorbance was measured at 550 nm and ADA activity calculated from the standard curve and expressed as U l^−1^ (1 U = 1 μmol product formed per min). For kinetic analyses, initial velocities were fitted by nonlinear regression to the Michaelis–Menten equation.

### Mitochondrial staining

Mitochondrial content was assessed using MitoTracker Green FM (MedChemExpress). Cells were stained with 1 μM MitoTracker Green FM for 25 min at 37 °C protected from light, washed with PBS and analyzed by flow cytometry. Mitochondrial content was quantified as MitoTracker Green in viable cells.

### IL-12 reporter assay

Activity of surface-displayed IL-12 was assessed using HEK-Blue IL-12 reporter cells (InvivoGen, hkb-il-12). Cells (5.4×10^4^ per well) were incubated with the indicated bacterial constructs for 36 to 48 hours. Culture supernatants were incubated with QUANTI-Blue reagent, incubated at 37 °C for 4 to 6 hours and secreted embryonic alkaline phosphatase activity was quantified by absorbance at 650 nm using a multiplate reader.

### *In vitro* cytotoxicity and lymphocyte functional assays

Primary human NK cells, MSLN CAR-NK cells or CD70 CAR-T cells were exposed to *E. coli* displaying ADA, IL-12, ADA–IL-12 or scaffold control at a bacteria-to-effector-cell ratio of 500:1 in the presence or absence of 10 mM adenosine (ADO), inosine (INO) or vehicle control. Tumor target cells were added at an effector-to-target (E:T) ratio of 1:1. Cocultures were performed in RPMI 1640 supplemented with 10% heat-inactivated FBS, 1% penicillin–streptomycin and 5 ng ml-1 IL-15. Cytotoxicity was assessed after 16 to 18 hours for OCI-AML3 and NOMO-1 cells and 24–28 hours for AsPC1 cells, with exact incubation times indicated in the corresponding figure legends. Target-cell viability was quantified by flow cytometry using a fixable viability dye and reporter expression to identify tumor cells and normalized to target-cell-only controls.

For analysis of degranulation and intracellular cytokine production, effector and target cells were cocultured under the same conditions for 6 hours. Anti-CD107a antibody was added at the start of coculture, and GolgiPlug (brefeldin A, BD Biosciences) was added after 1 hour, followed by incubation for an additional 4 to 6 hours. Cells were subsequently stained for surface markers and viability, fixed and permeabilized using Cytofix/Cytoperm (BD Biosciences), and stained intracellularly for IFN-γ and TNF. Degranulation and cytokine production were quantified within the respective NK- or T-cell populations by flow cytometry. Samples were acquired using a Sony ID7000 spectral flow cytometer.

### Flow-cytometric immunophenotyping

Primary human T and NK cells were cocultured with bacterial constructs at a 500:1 bacteria-to-immune-cell ratio for 24 hours in the presence or absence of 10 mM ADO or INO replenished every 8 to 12 hours. Cells were stained with viability, surface and, where indicated, intracellular markers. Antibodies, fluorophores, clones, and manufacturers are provided in Supplementary Table 2. Samples were acquired using a Sony ID7000 spectral flow cytometer and analyzed using FlowJo v10.10.1 (BD Biosciences), including FlowJo plugins for UMAP dimensionality-reduction analysis.

### Seahorse extracellular flux analysis

Primary human T and NK cells were cocultured with the indicated bacterial constructs for 24 hours in the presence of 10 mM adenosine (ADO), 10 mM inosine (INO) or vehicle DMSO control, replenished every 8 to 12 hours. Cells were subsequently washed and rested for 12 hours before metabolic analysis. Oxygen consumption rate (OCR) and extracellular acidification rate (ECAR) were measured using an XFe96 Extracellular Flux Analyzer (Agilent Technologies).

For metabolic profiling, 2.5×10^5^ cells per well were seeded onto poly-D-lysine-coated XFe96 microplates in XF RPMI medium (pH 7.4; Agilent) supplemented with 10 mM glucose, 2 mM glutamine and 1 mM pyruvate. Plates were centrifuged at 300 × g for 2 minutes and equilibrated for 1 hour at 37 °C in a non-CO_2_ incubator. OCR and ECAR were measured at baseline and following sequential compound injection using the Seahorse XF T Cell Metabolic Profiling Kit (Agilent). Basal and maximal respiration and spare respiratory capacity were calculated from OCR measurements.

For glycolytic profiling, 2.5 × 10^5^ cells per well were transferred onto poly-D-lysine-coated XFe96 microplates in XF DMEM medium (pH 7.4; Agilent) supplemented with 10 mM glucose, 2 mM glutamine and 1 mM pyruvate and equilibrated for 1 hour at 37 °C in a non-CO_2_ incubator. ECAR was measured using the Seahorse XF Glycolysis Stress Test Kit (Agilent). Sensor cartridges were hydrated overnight in XF calibrant (Agilent) at 37 °C in a non-CO_2_ incubator.

### Bulk RNA sequencing and analysis

Primary human T and NK cells from three independent donors were cocultured with engineered bacteria (500:1 bacteria-to-immune-cell ratio) for 72 hours. RNA was isolated using the RNeasy Mini Kit (QIAGEN) and subjected to paired-end sequencing on a NovaSeq X Plus platform (Plasmidsaurus). Reads were quality-filtered using fastp v0.24.0 and assessed using FastQC v0.12.1. Filtered reads were aligned to the hg38 reference genome using STAR v2.7.11b, followed by UMI-based deduplication using UMIcollapse v1.1.0. Gene-level expression was quantified using featureCounts (Subread v2.1.1). Differential expressions were analyzed using DESeq2 with donor as a blocking factor. Genes with ≥10 counts in at least three samples were retained, and *P* values were adjusted using the Benjamini–Hochberg method. For heatmaps, variance-stabilized expression values were corrected for donor-associated variation and row-scaled. For E. ADA–IL-12 versus E. scf, genes were selected at adjusted *P* < 0.10 and baseMean > 5 for T cells and adjusted *P* < 0.05 and baseMean ≥ 20 for NK cells. GSEA was performed using GSEApy v0.12 and MSigDB Hallmark gene sets.

### Animal experiments

Female BALB/c, female C57BL/6 mice and NOD.Cg-*Prkdc^scid^ Il2rg^tm1Wjl^*/SzJ (NSG) mice were maintained under pathogen-free conditions. Mice were monitored throughout the experiments and euthanized according to the humane endpoints specified in the approved animal protocol. Tumor growth was monitored by serial measurements, and survival was assessed until predefined humane endpoints were reached. Where indicated, separate cohorts were used for survival studies and mechanistic analyses.

### B16-F10 melanoma model

Female C57BL/6 mice were subcutaneously injected with 5×10^5^ B16-F10 melanoma cells. Mice received intratumoral injections of 20 μl of sterile PBS, E. scf, E. IL-12, E. ADA or E. ADA-IL-12 (0.5×10^9^ CFU per dose) on days 7, 11, 14 and 18 after tumor implantation. Treatment groups comprised *n*=3 mice for PBS and *n*=6 mice for E. scf, E. IL-12, E. ADA and E. ADA–IL-12. Tumor growth and survival were monitored until predefined humane endpoints were reached. Long-term survivors from the E. ADA (*n*=2 out of 6) and E. ADA–IL-12 (*n*=4 out of 6) groups were rechallenged with 5×10^5^ B16-F10 cells subcutaneously in the retro flank without further treatment and compared with age- and gender-matched naïve mice (*n*=3) to assess tumor growth and survival after rechallenging.

### Syngeneic WEHI-3B AML model

Female BALB/c mice were injected intravenously with 3×10^6^ NanoLuc/eGFP^+^-expressing WEHI-3B cells. Mice received intravenous PBS or 1×10^8^CFU of E. scf, E. ADA, E. IL-12 or E. ADA–IL-12 on days 5 and 10 after leukemia inoculation. Survival cohorts comprised *n*=7 mice per the treatment groups E. ADA, E. ADA–IL-12, E. scf and PBS control group and *n*=6 for E. IL-12-treated mice. For leukemic-burden, bacterial biodistribution, plasma cytokine and metabolite analyses and immune-profiling studies, mice were euthanized on day 14 after WEHI-3B inoculation in a separate experiment. Peripheral blood, bone marrow, spleen and liver were collected and cells isolated. These cohorts comprised *n*=4 mice per E. scf, E. ADA, E. IL-12 and E. ADA–IL-12 treatment group and *n*=3 PBS-treated mice. Leukemic burden was quantified by flow-cytometric detection of GFP^+^ WEHI-3B cells, and immune-cell populations were analyzed by flow cytometry. For bacterial biodistribution studies, peripheral blood, bone marrow, spleen and liver were collected 2 h and/or 4 d after the second bacterial administration, as specified for each experiment. Bacterial burden was quantified by colony-forming unit (CFU) analysis. Circulating cytokines were analyzed in separate cohorts at the indicated time points.

### AsPC1 xenograft and MSLN CAR-NK therapy

Male and female 6–8-week-old NSG mice were injected subcutaneously with 5×10^6^ AsPC1 cells. After tumor establishment, mice received two intravenous infusions of 5×10^6^ mesothelin (MSLN)-targeted CAR-NK cells, followed by intravenous PBS, E. scf or E. ADA–IL-12 (1×10^8^ CFU per dose) on days 18, 25, 32 and 39 after tumor implantation. Recombinant human IL-2 (75 kU per mouse) was administered intraperitoneally every other day. Tumor growth and survival were monitored in mice treated with PBS (n=3), E. scf (n=6) or E. ADA–IL-12 (n=7), with all groups receiving MSLN CAR-NK cells. A separate cohort was euthanized on day 36 for bacterial biodistribution and CAR-NK trafficking analyses. Two mice per treatment group were analyzed, with two tumors collected per mouse. Intratumoral CAR-NK cell abundance was quantified by flow cytometry as the percentage of GFP^+^ cells among human CD45^+^ cells. In a separate experiment, tumors were collected on day 28 for fluorescence microscopy.

### Orthotopic 4T1 mammary carcinoma model

Female BALB/c mice were orthotopically implanted with 0.5×10^6^ mCherry-expressing 4T1 cells into the mammary fat pad in 6 to 8-week-old mice. Mice received PBS, E. scf, 7.5 μg soluble protein ADA from calf intestine (Roche, 10102105001) plus 10 μg recombinant IL-12 (BioLegend, 577006) or E. ADA–IL-12 on days 6 and 9 intratumorally. Bacterial treatments were administered at 1 × 10^8^ CFU per dose. Tumor growth and survival were monitored in mice treated with PBS (n=5), E. scf (n=7), protein ADA plus protein IL-12 (n=7) or E. ADA–IL-12 (n=7). In a separate mechanistic cohort, tumors were collected on day 14, weighed and processed for flow cytometry (PBS and E. scf, n=3 mice per group; soluble ADA plus recombinant IL-12 and E. ADA–IL-12, n=4 mice per group).

### Murine tissue processing

Tumors and solid organs were minced and digested for 1 hour at 37 °C in antibiotic-free RPMI containing 5% FBS, 10 mM HEPES and 1 mg ml^−1^ collagenase IV. Digested tissues were passed through 70-μm strainers and subjected to erythrocyte lysis where required. Bone marrow was isolated from the femurs and tibiae by centrifugation. Bones were placed in a 0.5-ml microcentrifuge tube with a punctured bottom nested within a 1.5-ml collection tube, and bone marrow was collected into the outer tube by centrifugation. Spleen, bone marrow and peripheral blood were processed into single-cell suspensions and subjected to erythrocyte lysis where required before downstream analysis.

### Bacterial biodistribution

Blood, bone marrow, tumors and peripheral organs were collected aseptically at the indicated time points. Tissue was processed in antibiotic-free media. Solid tissues were weighed, homogenized and serially diluted onto selective LB agar containing 50 μg ml^−1^ kanamycin. Plates were incubated at 37 °C for 24 to 48 hours, colonies were counted and bacterial burden was quantified as CFU g^−1^ tissue or CFU ml^−1^ blood.

### Spectral flow cytometric immunophenotyping of murine cells

Cells were incubated and washed with flow cytometry buffer (PBS supplemented with 2% BSA) and stained with viability dye and the indicated surface-antibody panels. Intracellular and intranuclear staining was performed using the True-Nuclear Transcription Factor Buffer Set (BioLegend) according to the manufacturer’s instructions. Samples were acquired on a Sony ID7000 spectral flow cytometer and analyzed using FlowJo v10.10.1 . Antibodies, fluorophores, clones and manufacturers are listed in Supplementary Table S2. Gating strategies are provided in the Supplementary Information.

### Immunohistochemistry staining of bone marrow sections

Bone marrow samples were collected 10 d after WEHI-3B tumor-cell injection and 2h after bacterial administration. Samples were fixed in 4% paraformaldehyde (PFA) for 48 h in the dark, washed with PBS and transferred to 70% ethanol at 5 °C until further processing for pathology examination. Formalin-fixed, paraffin embedded femur specimens were sectioned at 4μm thickness. Sections were stained with monoclonal antibody against DYKDDDDK/FLAG (D6W5B; #14793 Sigma Aldrich) and rabbit polyclonal antibody against *E. coli* O/K serotypes (PA1-7213, ThermoFisher). Antibody binding was visualized using an HRP-based detection system with 3,3′-diaminobenzidine (DAB) as the chromogenic substrate, followed by hematoxylin counterstaining. Sections were subsequently imaged by light microscopy. The specificity and reactivity of the antibodies were validated and optimized on cell pellets of bacteria including native *E.coli* and corresponding genetically modified strains. Microscopic examination of mouse femurs from experimental conditions was performed at 500x magnification under oil immersion. Sections were examined microscopically for the presence of bacteria at ×500 magnification under oil immersion. Images were acquired at ×1,000 magnification using an Olympus BX46 microscope equipped with a DP27 camera.

### Immunofluorescence microscopy

Freshly collected tumors were embedded in optimal cutting temperature compound, frozen at −80 °C and cryosectioned by iHisto, INC. Frozen sections were washed three times with PBS, fixed in 4% PFA for 30 min in the dark and washed again. Sections were permeabilized in PBS containing 1% Triton X-100 for 30 min on ice, washed with PBS containing 0.2% Triton X-100 and blocked for 2 h on ice in blocking buffer (PBS containing 0.2% Triton X-100, 2 mg ml^−1^ BSA and 10% mouse serum). For visualization of intratumoral bacteria, sections were stained with FITC-conjugated anti-E. coli antibody (Abcam, ab30522; 1:200) overnight at 4 °C. This antibody was diluted in blocking buffer mentioned previously and incubated overnight at 4 °C. Afterwards, slides were washed three times with PBS containing 0.2% Triton X-100, stained with DAPI, followed by three times washes with the same buffer, post-fixed in 1% PFA for 10 min and mounted using EverBrite Mounting Medium with DAPI (Biotium, Cat. no. 23002).

### Multiplex cytokine analysis

Peripheral blood was collected before euthanasia into lithium-heparin tubes from WEHI-3B mice on day 14. Bone marrow was isolated by centrifugation from femurs and tibiae collected from euthanized mice on day 14. Plasma from peripheral blood and bone marrow was isolated by centrifugation at 4,000 × g for 20 min at 4 °C and stored frozen at −80 °C until analysis. Cytokine concentrations were measured by Eve Technologies using a mouse multiplex Luminex immunoassay. Analytes are indicated in the corresponding figure legends, and cytokine concentrations are reported as pg ml^−1^.

### Statistical analysis and reproducibility

Statistical analyses were performed using GraphPad Prism (version 11.1.1; GraphPad Software) and RStudio (version 2026.01.0, Build 392; Posit Software, PBC). Data are presented as mean±s.e.m. unless otherwise specified. Exact sample sizes (n), definitions of biological replicates, statistical tests and error-bar definitions are provided in the corresponding figure legends. No statistical method was used to predetermine sample size. Mice were randomized to treatment groups after tumor establishment where applicable; investigators were not blinded to treatment allocation or outcome assessment.

For comparisons between two groups, two-sided paired or unpaired Student’s t-tests or two-sided Mann–Whitney U tests were used as appropriate to the experimental design and data characteristics. Donor-matched experiments using primary human cells were analyzed using paired tests. Comparisons involving multiple groups were analyzed using one-way ANOVA or, for experiments with two independent variables, two-way ANOVA, with repeated-measures analyses used for matched observations where appropriate. Multiple comparisons were corrected using Dunnett’s, Tukey’s, Šídák’s, Holm–Šídák’s or Bonferroni’s procedures, as specified in the corresponding figure legends. Associations between continuous variables were assessed by Pearson’s correlation analysis. Survival distributions were estimated using the Kaplan–Meier method and compared using two-sided log-rank (Mantel–Cox) tests.

For RNA-sequencing analyses, differential gene expression was analyzed in R and P values were adjusted for multiple testing using the Benjamini–Hochberg method. Gene set enrichment analyses (GSEA), volcano plots and gene expression heatmaps were performed or generated in R as described in the corresponding Method sections.

All statistical tests were two-sided. Exact P values are reported in the corresponding figures and tables. For t-tests and ANOVAs, test statistics (t and F, respectively) and degrees of freedom are reported where applicable. The number of independent experimental repetitions is specified in the corresponding figure legends.

## Supplementary Material

This is a list of supplementary files associated with this preprint. Click to download.

- ExtendedData.pdf
- SupplementalData.pdf

## Figures and Tables

**Figure 1.**
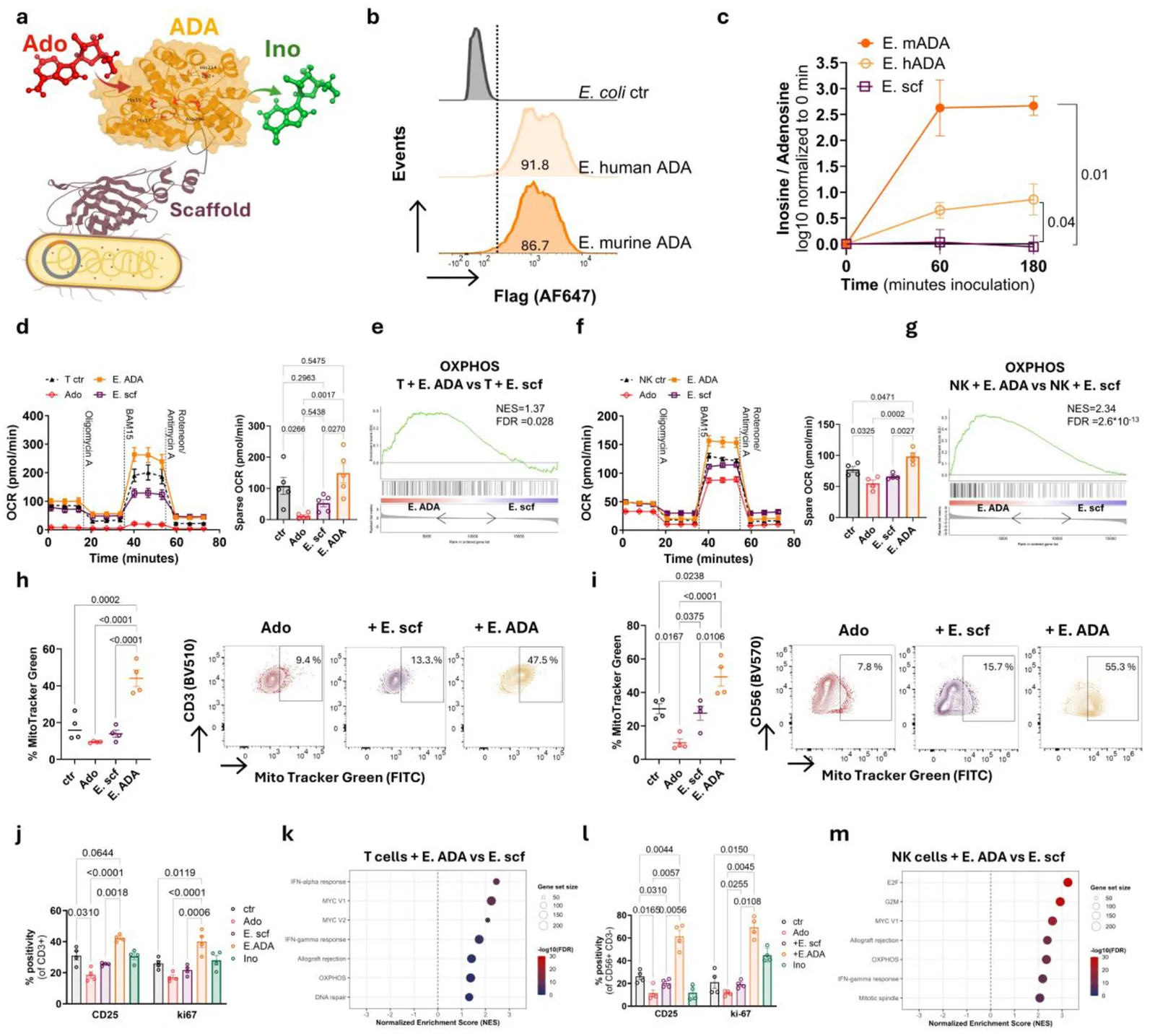
*E. coli*-displaying adenosine deaminase (E. ADA) reverses adenosine-mediated metabolic suppression. **a,** *E. coli* displaying scaffold YiaT181 fused to ADA to convert adenosine (ADO) to inosine (INO). Created with BioRender.com. Structure prediction was performed with AlphaFold v.2 and visualized in PyMOL. **b,** Representative histograms of surface-exposed FLAG-tagged scaffold YiaT181 displaying murine ADA (mADA) or human ADA (hADA), with FLAG-negative bacteria control. **c,** ADA activity of YiaT181-mADA or YiaT181-hADA measured by targeted LC–MS as log10-transformed inosine-to-adenosine (INO/ADO) ratio after 0, 60 and 180 min. **d**,**f**, Oxygen-consumption-rate (OCR) profiles and spare respiratory capacity of T (**d**) and NK cells (**f**) treated with ADO, ADO + E. scf or ADO + E. ADA. Oligomycin, BAM15 and rotenone/antimycin A injections are marked. Spare respiratory capacity was calculated as maximal minus basal OCR. **e**,**g**, Hallmark OXPHOS enrichment curves in T cells (**e**) and NK cells (**g**) treated with E. ADA versus E. scf supplemented with ADO. **h**,**i**, Mitochondrial abundance measured by MitoTracker Green staining in T- (**h**) and NK cells (**i**). **j**,**l**, Surface expression level of CD25 and intranuclear Ki-67 in T cells (**j**) and NK cells (**l**). **k**,**m**, Enriched Hallmark gene sets in E. ADA versus E. scf + ADO in T cells (**k**) and NK cells (**m**). Bubble size indicates gene-set size, color indicates log10(FDR); FDR, false discovery rate. Data are mean±s.e.m. of n=3 (**c**) independent biological replicates and n=3 (**e**,**g**,**k**,**m**), n=4 (**f**,**h-j**,**l**) and n=5 (**d**) independent donors. Each point represents an independent biological replicate or donor. (**d**,**f**,**h**,**i**) One-way ANOVA and (**c**,**j**,**l**) two-way ANOVA with Tukey’s test.

**Figure 2.**
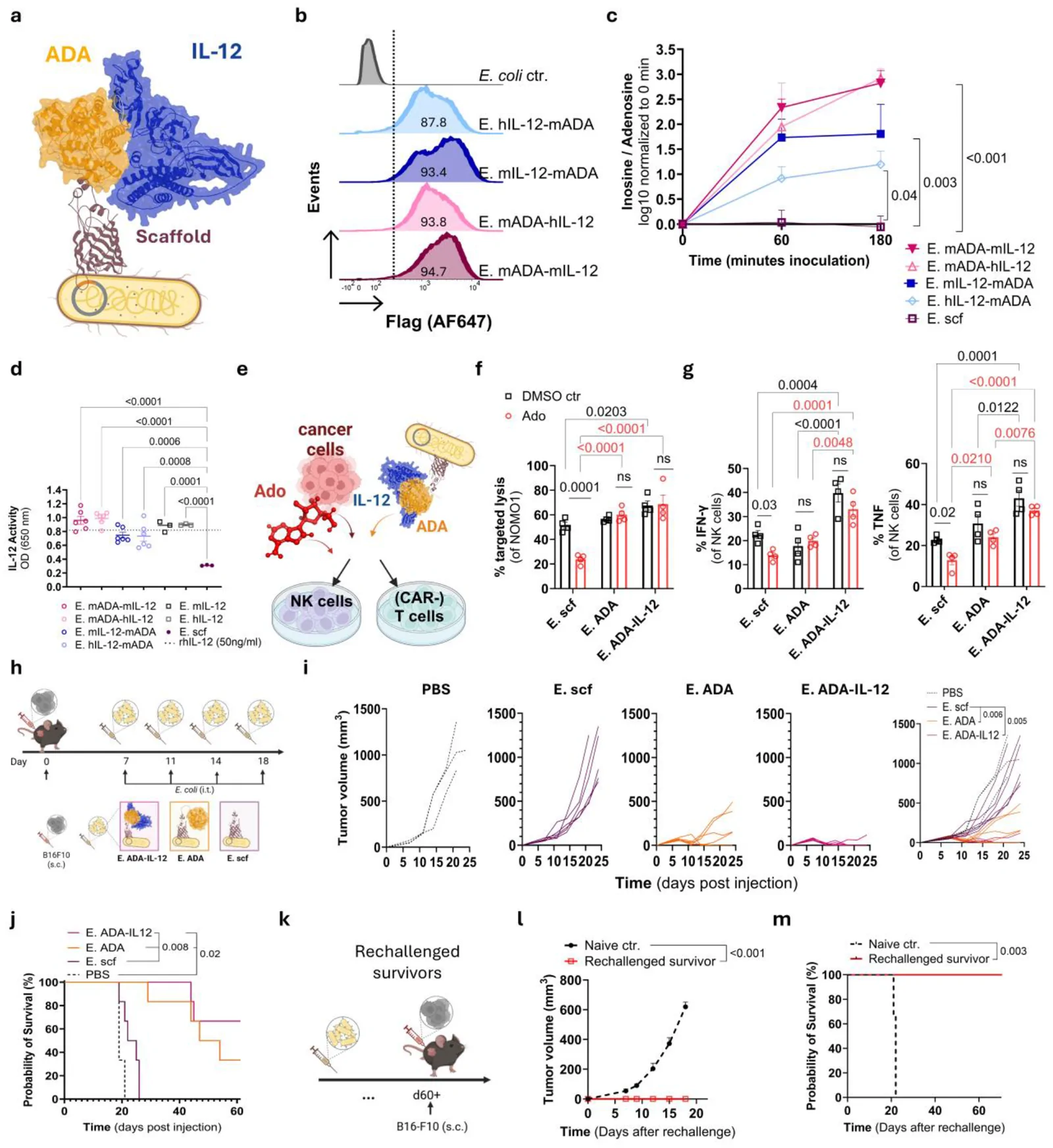
IL-12 incorporation into the *E. coli-*displayed ADA (E. ADA–IL-12) enhances antitumor activity. **a,** *E. coli* displaying YiaT181-scaffold attached ADA–IL-12 dual protein. Created with BioRender.com. Structure prediction with AlphaFold v.2, visualized in PyMOL. **b,** Flow-cytometric analysis of FLAG-tagged reciprocal fusions of murine ADA (mADA) with human or murine IL-12 and FLAG-negative bacteria control. **c,** ADA activity of the indicated ADA–IL-12 fusion constructs measured by targeted LC–MS as the log10-transformed inosine-to-adenosine (INO/ADO) ratio at 0, 60 and 180 min. **d,** IL-12 bioactivity of the indicated E.ADA–IL-12 constructs measured using HEK-Blue IL-12 reporter cells. **e,** Schematic of the *in vitro* coculture assay of NK- or T cells with target cancer-cells in presence of ADO and *E. coli* displaying ADA–IL-12 dual constructs. Created with BioRender.com. **f**,**g**, NK cell-mediated tumor cell lysis (**f**) and intracellular IFN-γ and TNF expression (**g**) upon coculture with NOMO-1 cells (E:T, 1:1) in presence of 10 mM ADO or DMSO (ctr.) and bacterial constructs as indicated. **h**, C57BL/6 mice were subcutaneously injected with 5×10^5^ B16-F10 melanoma cells and treated intratumorally (i.t.) with 5×10^8^ CFU E. scf, E. ADA, E. ADA–IL-12 (each n=6) or PBS (n=3) on days 7, 11, 14 and 18 after tumor implantation. **i**, Individual and grouped tumor growth curves and **j,** Kaplan–Meier survival analysis in B16-F10 mice. **k,** long-term survivors (>60 days) of E. ADA (n=2) and E. ADA–IL-12 (n=4) treated mice and three naïve controls were rechallenged with 5×10^5^ B16-F10 cells. **l,** Tumor growth and **m,** Kaplan–Meier survival following rechallenge. **c**,**d**,**f**,**g** Mean±s.e.m. of (**c**) n=3 and (**d**), n=6, independent biological replicates and (**f**,**g**) n=4 independent donors. **d**, One-way ANOVA with Dunnett correction; **c**,**f**,**g**,**i**, Two-way ANOVA with Tukey’s test and **l**; Šídák’s test; **j**,**m**, Log-rank (Mantel–Cox) test.

**Figure 3.**
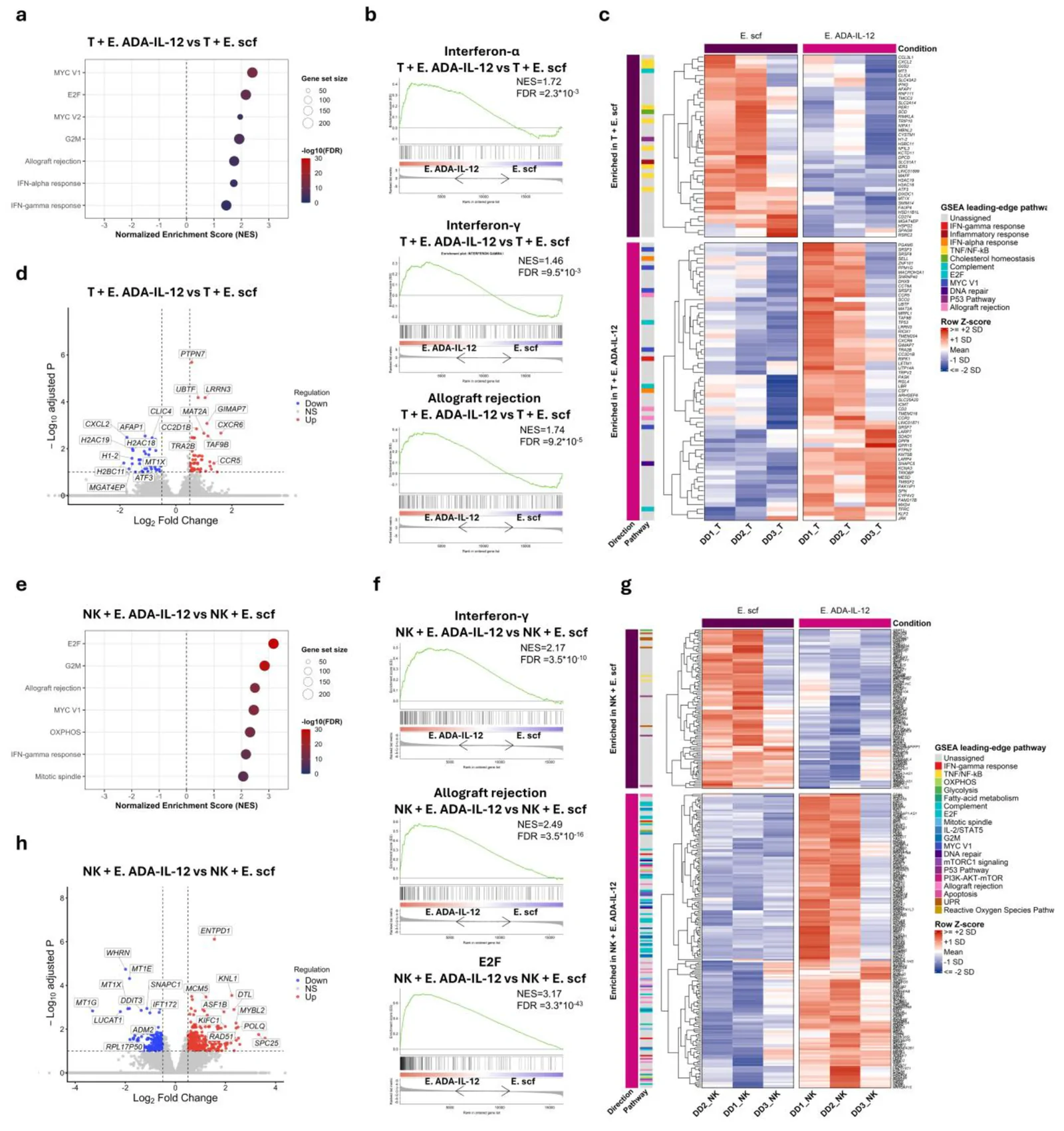
E. ADA–IL-12 promotes inflammatory transcriptional signatures in T and NK cells. Transcriptomic profiling of T cells (**a-d**) and NK cells (**e-h**) from healthy human donors (n=3) following co-culture with bacterial constructs displaying ADA–IL-12, ADA, and scaffold in presence of adenosine (10 mM); panels show E. ADA–IL-12 compared with E. scf. **a,** Hallmark gene-set enrichment analysis (GSEA) in T cells. **b,** GSEA enrichment plots for IFN-α and IFN-γ, and allograft rejection in T cells. **c,** Heatmap of differentially expressed genes in T cells (adjusted *P* <0.10; baseMean ≥5), with corresponding Hallmark pathway annotations. **d,** Volcano plot of differential gene expression in T cells. **e,** Hallmark GSEA in NK cells. **f,** GSEA enrichment plots for IFN-γ response, allograft rejection and E2F targets in NK cells. **g,** Heatmap of differentially expressed genes in NK cells (adjusted *P* <0.05; baseMean ≥20), with corresponding Hallmark pathway annotations. **h,** Volcano plot of differential gene expression in NK cells. Heatmaps show donor-corrected, row-scaled expression (Z-score). Positive normalized enrichment scores (NES) indicate enrichment in E. ADA–IL-12 and negative NES enrichment in E. scf. FDR, false discovery rate.

**Figure 4.**
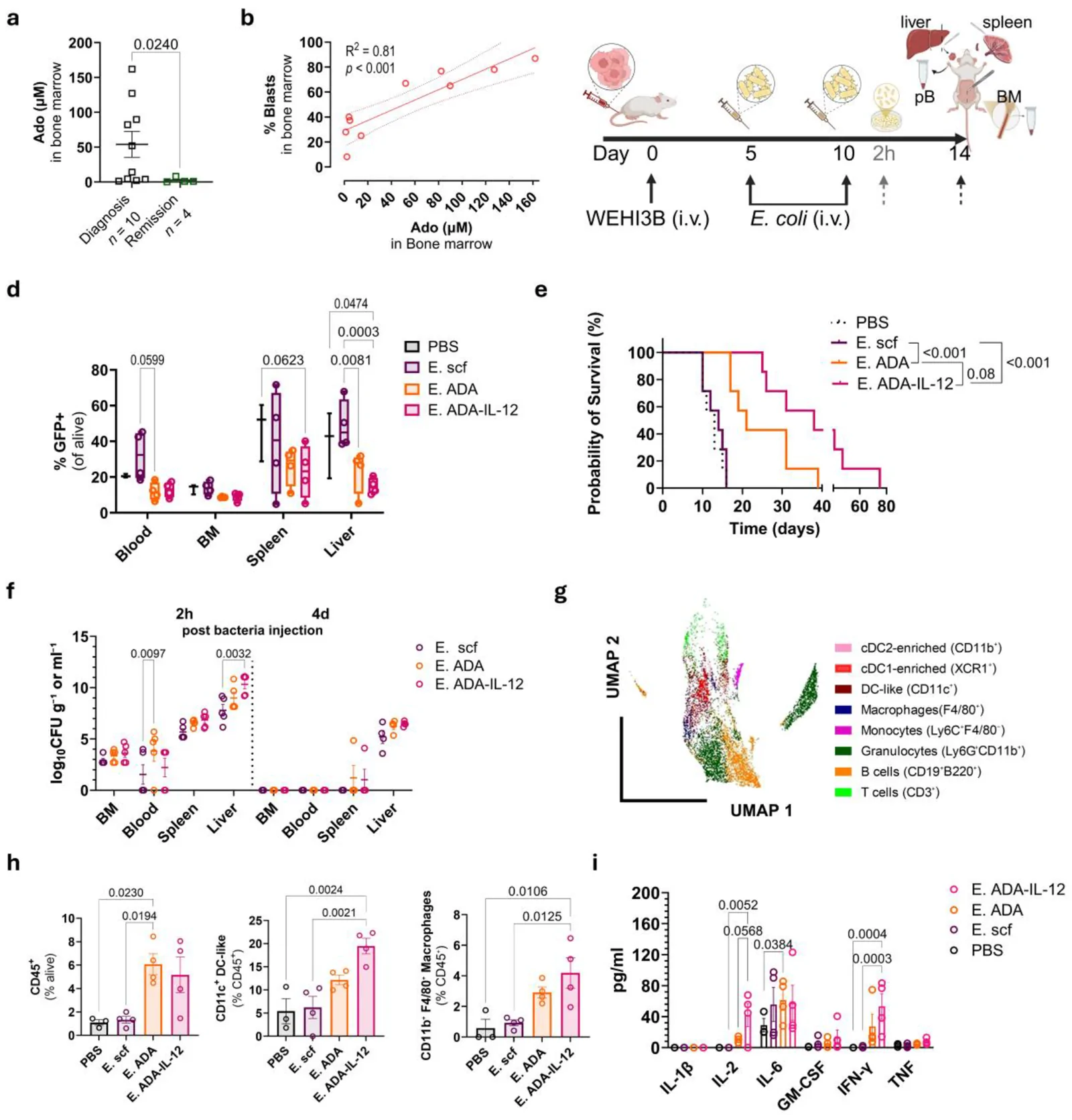
Targeting adenosine-rich AML with E. coli ADA–IL-12 promotes immune remodeling and prolongs survival. **a**,**b**, Adenosine level quantified by LC-MS in bone marrow (BM) samples from patients with AML at diagnosis (n=10) versus remission (n=4) (**a**) and correlated with blast percentage (**b**). **c**, Syngeneic BALB/c mice received 3×10^6^ WEHI-3B cells intravenously on day 0. Mice received 1×10^8^ CFU of E. scf, E. ADA, E. ADA–IL-12 or PBS on days 5 and 10. Created with BioRender.com. **d,** Leukemic burden in blood, BM, spleen, and liver was quantified by flow cytometry of GFP^+^ WEHI-3B cells on day 14. **e,** Kaplan-Meier survival analysis in WEHI3B-bearing mice treated with E. ADA–IL-12, E. ADA, E. scf, or PBS (n=7 per group). **f,** Biodistribution of E. scf, E. ADA, E. ADA–IL-12 in blood, BM, spleen and liver 2 hours and 4 days after day 10 bacteria injection, quantified as colony-forming units (CFU) per ml blood or gram (g). **g**, Uniform manifold approximation projection (UMAP) of concatenated hepatic CD45^+^ cells across treatment groups visualizing lineage-defined immune populations identified by multiparameter flow cytometry. **h,** CD45^+^, CD11c^+^ dendritic cell-like and CD11b^+^F4/80^+^ macrophage-enriched cell populations in hepatic tissue, day 14 post tumor injection by flow-cytometry. **i**, Blood plasma cytokine level, day 14, assessed by Luminex multiplex immunoassay. Mean±s.e.m. (**a**,**f**,**h**,**i**) of **a,** bone marrow samples from n=14 patients diagnosed with AML and (**f**,**h**,**i**) tissue samples from *n*=4 (E. ADA–IL-12, E. ADA, E. scf) and *n*=3 (PBS) individual mice per group; **a**, two-tailed Mann-Whitney U test; **b**, Pearson correlation; **d**,**f**,**i**, Two-way ANOVA with Tukey’s test; **e**, Log-rank (Mantel–Cox) test; **h,** One-way ANOVA with Tukey’s test.

**Figure 5.**
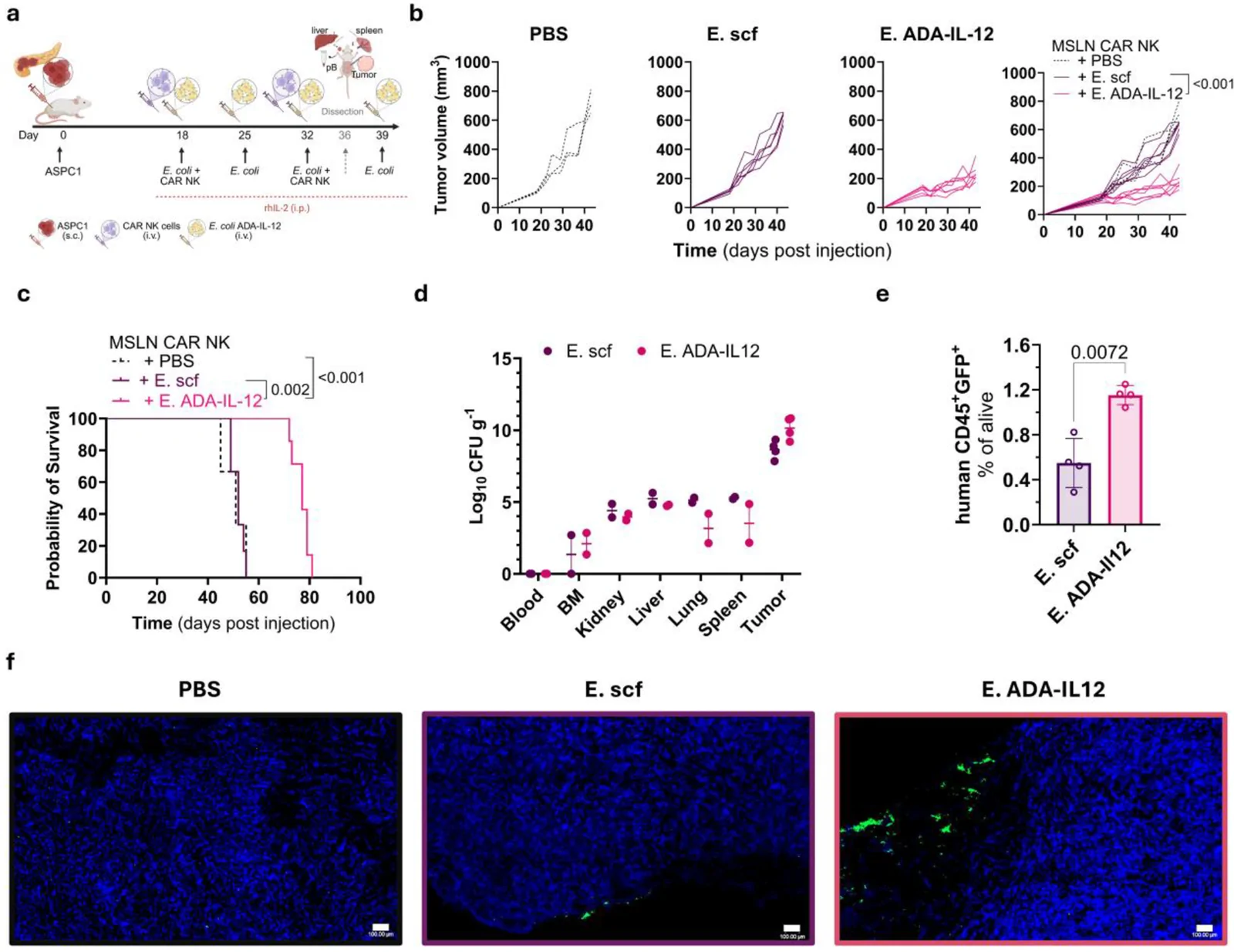
E. ADA–IL-12 enhances CAR-NK cell therapy in a pancreatic cancer xenograft model. **a,** NSG mice received AsPC1 pancreatic ductal adenocarcinoma (PDAC) cells followed by intravenous (i.v.) E. ADA–IL-12, E. scf or PBS on days 18, 25, 32 and 39, and two mesothelin (MSLN)-targeted CAR-NK cell infusions (5×10^6^) on days 18 and 32, accompanied by intraperitoneal (i.p.) IL-2 every other day. **b,** Individual and grouped tumor growth curves over time and **c,** Kaplan-Meier survival analysis of mice treated with MSLN-CAR NK cells and PBS (n=3), E. scf (n=6), or E. ADA–IL-12 (n=7), i.v. **d,** Biodistribution of E. scf and E. ADA–IL-12 in blood, tumor, spleen and liver on day 36, quantified as colony-forming units (CFU) per gram tissue. **e,** Intratumoral MSLN CAR-NK cells on day 36, quantified by flow cytometry as the percentage of GFP^+^ cells among human CD45^+^ cells. **f,** Representative fluorescence microscopy images showing bacterial localization (green) in tumors of mice treated with PBS, E. scf. or E. ADA–IL-12 with MSLN CAR NK cells on day 28 post tumor implantation. DAPI-stained nuclei (blue). Scale bars (white) 100 μm. **d**,**e**, Mean±s.e.m. of **d**,**e**, n=4 tumors from n=2 mice per group. **c**, Log-rank (Mantel–Cox) test; **d,** Two-way ANOVA with Šídák’s test; **e,** two-sided unpaired Welch’s *t*-test.

**Figure 6.**
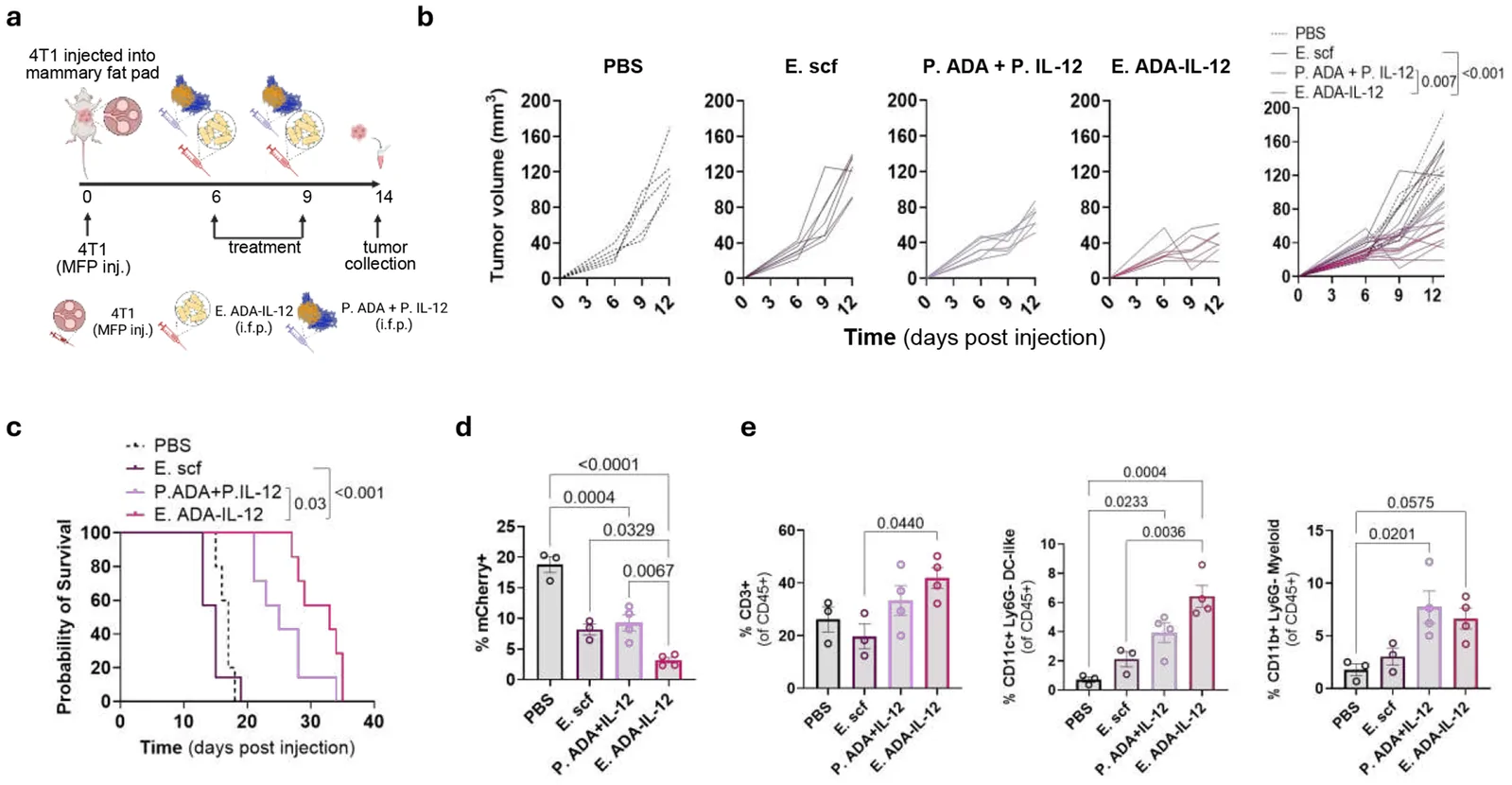
E. ADA–IL-12 induces potent tumor control in an orthotopic 4T1 breast cancer model. **a,** 4T1 cells were orthotopically implanted into the mammary fat pad, followed by 1×10^8^ CFU E. ADA–IL-12, E. scf, PBS or soluble protein ADA plus recombinant IL-12 on days 6 and 9. **b,** Tumor growth over time and **c,** Kaplan-Meier survival analysis of mice treated i.t. with PBS (n=5), E. scf (n=7), E. ADA–IL-12 (n=7), or protein ADA plus IL-12 (P. ADA + P. IL-12, n=7). In a separate experiment, tumors were collected on day 14 for endpoint analyses of **d**, mCherry^+^ cells quantified by flow cytometry and **e**, Tumor-infiltrating CD3^+^ T cells, CD11c^+^ DC-like and CD11b^+^ Myeloid cells in mice injected with PBS and E. scf. (n=3, both) and E. ADA–IL-12 or P. ADA + P. IL-12 (n=4, both). **d**,**e**, One-way ANOVA with Tukey’s test; **b**, Two-way ANOVA with Tukey’s test; **c**, Log-rank (Mantel–Cox) test.

## Data Availability

All data generated during this study are available within the paper. The bulk RNA-seq data will be deposited to the Gene Expression Omnibus (GEO) and the corresponding accession number will be provided upon availability. Source data will be provided as accompanying Source Data files with this paper before publication.

## Code availability

Code used for RNA-sequencing and associated computational analyses will be made available..
